# Expression of hemagglutinin protein from the avian influenza virus H5N1 in a baculovirus/insect cell system significantly enhanced by suspension culture

**DOI:** 10.1186/1471-2180-6-16

**Published:** 2006-02-24

**Authors:** Nitar Nwe, Qigai He, Sudarat Damrongwatanapokin, Qingyun Du, Ivanus Manopo, Yukol Limlamthong, Beau James Fenner, Lynn Spencer, Jimmy Kwang

**Affiliations:** 1 Animal Health Biotechnology Group, Temasek Life Science Laboratory, 1 Research Link, National University of Singapore, Singapore 117604, Singapore; 2 Department of Livestock Development, National Institute of Animal Health, Chatuchak, Bangkok, Thailand

## Abstract

**Background:**

Prevention of a possible avian influenza pandemic necessitates the development of rapid diagnostic tests and the eventual production of a vaccine.

**Results:**

For vaccine production, hemagglutinin (HA1) from avian influenza H5N1 was expressed from a recombinant baculovirus. Recombinant HA1 was expressed in monolayer or suspension culture insect cells by infection with the recombinant baculovirus. The yield of rHA1 from the suspension culture was 68 mg/l, compared to 6 mg/l from the monolayer culture. Immunization of guinea pigs with 50 μg of rHA1 yielded hemagglutinin inhibition and virus neutralization titers of 1:160 after two times vaccination with rHA1 protein.

**Conclusion:**

Thus, the production of rHA1 using an insect suspension cell system provides a promising basis for economical production of a H5 antigen.

## Background

Influenza A viruses have a segmented genome of single-stranded negative-sense RNA and belong to the family *Orthomyxoviridae *[1]. They have been isolated from a variety of animals, including humans, pigs, horses, sea mammals, and birds [2]. There are 16 subtypes of influenza virus A, among them the highly pathogenic avian H5N1 viruses which caused 18 confirmed infections and six deaths in Hong Kong in 1997. This virus can apparently be transmitted directly from birds to humans with no intermediate mammalian host [3]. Infection of poultry with highly pathogenic avian influenza virus can be devastating in terms of flock morbidity and mortality, economic loss and social disruption [4]. Swayne et al. [5] suggested that vaccination has the potential to reduce environmental contamination with avian influenza virus and prevent subsequent bird-to-bird transmission [5]. In order to prevent spread of influenza viruses, emphasis must be placed on biosecurity and flock management practices, the development of rapid diagnostics [4] and vaccine production [6]. Current influenza vaccines include a subunit vaccine [7-10], attenuated vaccine [11,12], DNA vaccine [13] and inactivated influenza vaccine [14], with the latter being the most widely used on a commercial scale [6].

Current inactivated vaccines production requires large numbers of embryonated chicken eggs [9]. The major problems for production of this type of vaccine are the requirements for high-level biocontainment facilities, and in some cases, an inability to obtain high yields of virus in embryonated chicken eggs [6]. The yield of total viral protein from the 10-day-old chicken embryos is typically 20–40 mg/200 eggs [15]. Therefore large scale preparation of inactivated vaccine by this method is not feasible.

Subunit vaccines, recombinant hemagglutinin and neuraminidase [7-10] may be an attractive alternative to the inactivated vaccine, although none are currently in use as commercial vaccines. The preparation of such vaccines is obviously safer than for an inactivated vaccine and preparation of candidate subunit vaccines is simple [16]. Moreover, subunit vaccines do not generate antibody responses to internal influenza viral proteins and thus allow distinction between vaccinated and infected birds [8].

Hemagglutinin protein is the receptor-binding and membrane fusion glycoprotein of influenza virus and the target for infectivity-neutralizing antibodies [17,18]. The entire hemagglutinin protein (HA) from the H5N1 is composed of 568 amino acids, with a molecular weight of 56 kDa. The HA molecule consists of HA1 and HA2 subunits, with the HA1 subunit mediating initial contact with the cell membrane and HA2 being responsible for membrane fusion [17].

The recombinant hemagglutinin protein can be produced in different ways such as expression of hemagglutinin protein in the insect cell system [8-10,16,19,20] or in the recombinant baculovirus express in insect larvae [21]. Previous baculovirus/insect cell systems have been used to express hemagglutinin genes isolated from avian influenza subtypes. However, a protein band corresponding to rHA1 from baculovirus infected cells was not observed by SDS/PAGE of total cell protein. This could have been due to a low level of expression or alternatively incorrect glycosylation of the polypeptide in insect cells [19].

The recombinant baculovirus/insect cell system can be carried out under monolayer and suspension culture. In this research the HA1 gene from avian influenza virus H5N1 encoded recombinant baculovirus was constructed and infected into insect cells under different culture methods and condition. The expression level of recombinant hemagglutinin protein in baculovirus/insect cell system was compared. Guinea pigs were immunized with recombinant hemagglutinin protein in order to evaluate its immunogenicity.

## Results and discussion

### Expression of recombinant hemagglutinin in insect cells under monolayer and suspension culture conditions

A recombinant baculovirus carrying the HA1 gene of avian influenza A/goose/Guangdong/97 (H5N1) was constructed and propagated in Sf9 cells. After 4 generations, the virus stock was collected and the expression level of recombinant hemagglutinin protein was determined under monolayer and suspension culture conditions.

**Figure 1:**
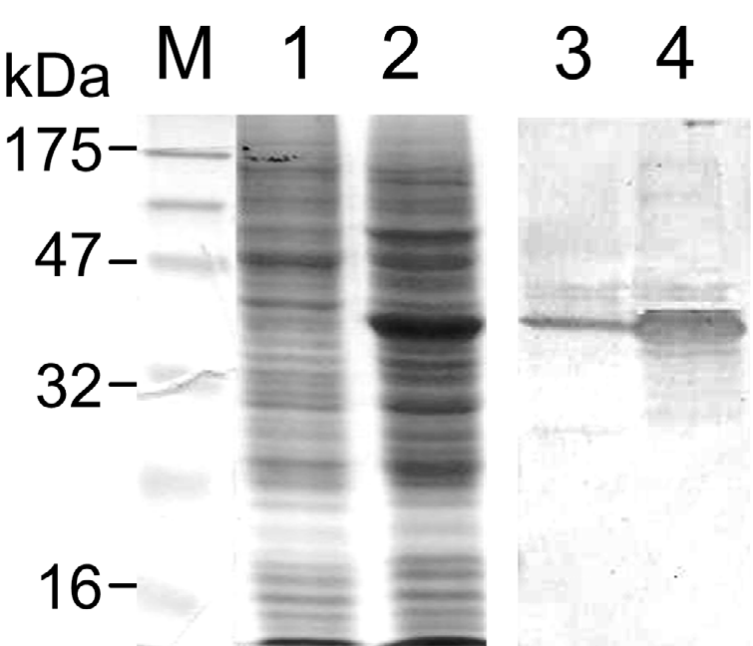
SDS-PAGE and immunoblot analysis of rHA1 protein expressed in a baculovirus/insect cell system under monolayer (lanes 1 and 3) and suspension (lanes 2 and 4) culture conditions. Values on the left indicate molecular weights of the marker protein (lane M). The rHA1 was detected by immunoblotting with rabbit anti-H5N1 serum.

**Figure 2:**
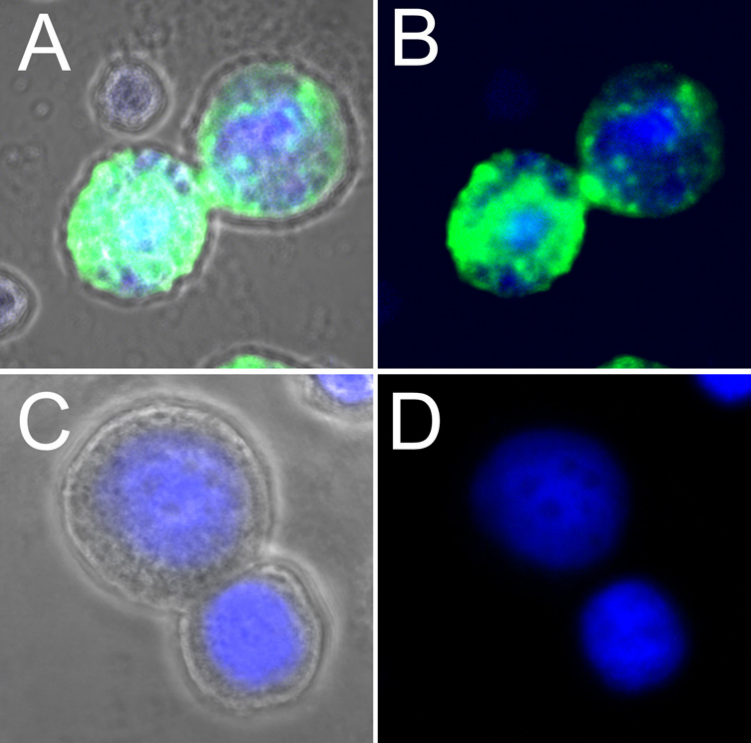
Confocal imaging of recombinant baculovirus infected Sf9 cell (A and B) and uninfected Sf9 cell (C and D). DAPI was used to stain cell nuclei and monoclonal antibody specific to HA1 of avian influenza H5 was used to localize rHA1 in recombinant baculovirus infected cells and uninfected cells.

The Sf9 cells were infected with the recombinant baculovirus and cultured in 175 cm^2 ^flasks for monolayer culture and in 500 ml baffled glass flasks for suspension culture. The monolayer culture was incubated in the incubator and the suspension culture was incubated in the shaking incubator for 0, 63, 87, 111 and 135 h. SDS-PAGE and immunoblot analysis of baculovirus infected Sf9 cells from monolayer and suspension cultures revealed an obvious difference in the amount of the 35 kDa rHA1 protein between the different cultures, with a far yield being obtained from the suspension culture than the monolayer culture after a 111 h incubation (Figure 1, compare lanes 1 and 2, and lanes 3 and 4). Confocal imaging of the rHA1-expressing Sf9 cells using a H5 influenza-specific monoclonal antibody indicated that the protein was localized primarily in the cytoplasmic space (Figure 2).

**Figure 3:**
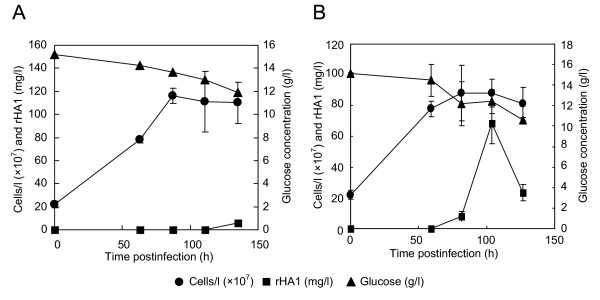
Expression of rHA1 in baculovirus infected Sf9 cells under monolayer (A) and suspension (B) culture conditions. Each data point is the average from triplicate experiments and the error bars represent the 95% confidence level.

Under monolayer culture conditions, a cell density of 10^9 ^cells/l yielded 6 mg/l of hemagglutinin protein (Figure 3A). In contrast, the suspension cell culture could be grown in reusable 500 ml baffled glass flasks with approximately the same cell density yielded almost 68 mg/l of rHA1 (Figure 3B). This may be due to a reduced efficiency of baculovirus entry into the Sf9 cells and lowered cell substrate uptake in the monolayer culture, as suggested in the Dee and Shuler [22] mathematical treatment of the diffusion-limited attachment rate in suspension and monolayer cultures.

### Influence of harvesting time on hemagglutinin expression in suspension culture

The Sf9 medium contains a mixture of sugars: glucose, fructose and sucrose. Glucose concentration in the culture remained above 10 g/l at the end of fermentation, indicating that the carbon source was not a limiting factor in this medium (Figure 3B). Suspension cultures were incubated in a shaker incubator, so optimization of harvesting time could be performed with ease in the shortest possible time. The results from the suspension culture are presented in Figure 3B, illustrating the cell growth pattern after infection and the kinetics of hemagglutinin protein in the infected Sf9 cells. There was an initial increase in the volumetric yield of hemagglutinin protein at the peak cell density, the maximum viable cell density postinfection. The observed hemagglutinin protein was 68 mg/l at 111 h post infection. At the very late phase (138 h) the yield of hemagglutinin protein in the cell decreased. The resultant secreted protein in the medium was 259 mg/l after 138 h compared with 174 mg/l at 111 h. This may be due to the lysis of some cells releasing occluded virus and some intracellular protein into the extracellular fluid. Possee [19] also reported that HA activity peaked at 24 h and decreased at 48 h postinfection in a similar expression system. In that study it was assumed that this decline presumably reflected cell lysis and subsequent degradation of HA. According to these results, appropriate harvesting time of baculovirus infected Sf9 cells was an important factor in obtaining a high yield of hemagglutinin.

Sugiura et al. [21] obtained 0.4–4 μg/larvae of purified hemagglutinin protein from the recombinant baculovirus infected in silkworm larvae. Possee [19] observed the hemagglutinin presented in the plasma membrane of the infected cells and HA activity of 6.5 × 10^2 ^HAU/10^6 ^cells was reached at peak infection of recombinant baculovirus to the *S. frugiperda *cells. In this study a yield of 77 μg/10^6 ^cells was obtained from the recombinant baculovirus infected in the Sf9 cells. This may be due to the expression of hemagglutinin protein localized in the cytoplasm of infected cells.

### Purification of hemagglutinin protein from the recombinant baculovirus infected Sf9 cell

**Figure 4:**
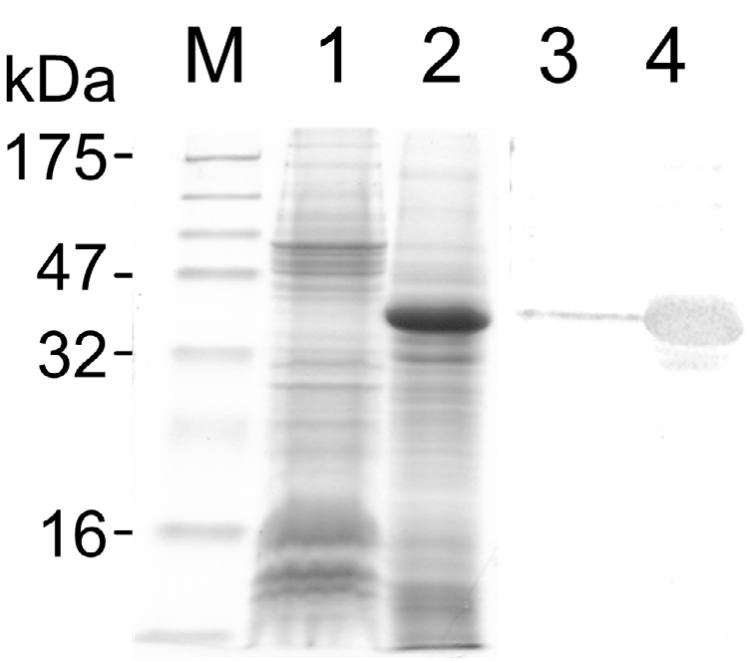
SDS-PAGE and immunoblot analysis of supernatant (lane 1 and 3) and pellet (lane 2 and 4) after lysis of recombinant baculovirus infected Sf9 cell with lysis buffer containing 1% NP-40. Values on the left indicate molecular weights of the maker protein (LaneM).

The expressed hemagglutinin protein was partially purified with the lysis buffer incorporated with 1% NP-40. Samples were taken from each purification step and analyzed by SDS-PAGE and immunoblotting (Figure 4). The majority of rHA1 presented as insoluble material in the pellet as evidenced by immunoblot analysis. Therefore recombinant protein is expressed as a dimeric species that is present in both insoluble and free forms in the infected Sf9 cells. When the cell lysis experiment was repeated under various pH conditions, no additional extraction was achieved and the protein of interest remained insoluble.

### Immunogenicity of recombinant hemagglutinin subunits in guinea pigs

There are numerous studies of the immune response to baculovirus-expressed recombinant hemagglutinin expressed in mice [9,23], chickens [8], horses [21] and humans [16,20]. Doses of 15–135 μg of rHA1 protein are typically required to induce the same level of immunity in humans as that achieved with a standard dose of inactivated influenza vaccine containing 15 μg of HA antigen [16]. Jones et al. [23] reported that higher doses of subunit vaccine were needed to induce responses comparable to those elicited by current licensed egg-derived, detergent-split influenza vaccines.

In order to study the immunogenicity of the recombinant hemagglutinin obtained from the suspension culture, guinea pigs were immunized with rHA1 as insoluble and detergent-solubilized protein and the immune response measured by hemagglutinin inhibition, HI and virus neutralization assays. The antibody response to these proteins is shown in Table 1. Neutralizing antibody responses to a titer of 1:160 and an HI titre of 1:160 were obtained after two vaccinations with 50 μg of insoluble rHA1 (Table 1). However, low HI titers and negative virus neutralization were observed in sera derived from guinea pigs immunized with the same dosage of detergent-solubilized recombinant hemagglutinin protein.

## Conclusion

These results show that recombinant hemagglutinin expressed from baculovirus elicits an antibody response in guinea pigs and induces HI and neutralization titers against to the H5N1 avian influenza virus. Therefore, this protein has good potential for use as antigen for subunit vaccine and diagnostic test. This system coupled with suspension culture could probably be easily adapted to large scale manufacturing of H5 antigen for the development of subunit vaccine and diagnostic test for H5 avian influenza virus.

## Methods

### Influenza virus H5N1 and rabbit anti H5N1

A/goose/Guangdong/97 (H5N1) virus was obtained from the allantoic fluid. Rabbits were injected intramuscularly with 1 mg of purified H5N1 virus in Freund's complete adjuvant (Sigma). Three successive injections were performed with 2-week intervals. Blood samples were collected 2 weeks after the last injection for serum preparation.

### Preparation of recombinant baculovirus

Influenza virus A/goose/Guangdong/97 (H5N1) was used to inoculate 10-day-old specific-pathogen-free (SPF) embryonated chicken eggs. Allantoic fluid containing the virus was harvested at day 14 and inactivated with 0.4% paraformaldehyde. RNA was extracted from the inactivated virus using TRIzol reagent (Invitrogen) and the HA1 gene amplified by RT-PCR using HA1 forward (5'-CGGGATCCGATGGAGAAAACAGTGCTTC-3') and reverse (5'-ACGCGTCGACTTTCACATATTTGGGGCATTC-3') primers. The amplified HA1 DNA was resolved on an agarose gel, purified (Qiaquick, Qiagen) and sequenced and ligated to the pFastBac (Invitrogen) donor vector. The resulting recombinant vector was introduced into the target bacmid by homologous recombination as described in the pFastBac protocol.

### Insect cell culture

Sf9 cells (Invitrogen) were transfected with recombinant bacmid DNA and the recombinant baculovirus collected from the culture medium at 72 h post-transfection as suggested by the manufacturer. Cells were maintained in monolayer cultures at 27°C. Cells were subcultured every 2 days by diluting seed cultures at 4.75 × 10^5 ^cells/ml to a cell density of 2.38 × 10^5 ^cells/ml with fresh medium. Transfection and recovery of recombinant baculovirus from Sf9 cultures was performed according to the pFastBac protocol (Invitrogen), and virus infectivity titer was determined on sample supernatants using a modified indirect immunofluorescence assay [24,25].

### Expression of recombinant hemagglutinin in insect monolayer and suspension cultures

Sf9 cells were grown as monolayers at 27°C in 175 cm^2 ^flasks using Sf900 II medium. Infection with recombinant baculovirus was at a multiplicity of infection of 0.4 PFU/cell using an initial cell density of 2.2 × 10^5 ^cells/ml. Cells were harvested at various times postinfection. For suspension culture, cells were grown in 100 ml volumes in 500 ml baffled glass flasks and incubated in a rotary shaker at 80 rpm and 28°C. The initial cell density was 2.2 × 10^5 ^cells/ml. The cultures were infected with the recombinant baculovirus with a multiplicity of infection of 0.4 PFU/cell and cells harvested from the culture medium at various times postinfection. Cells were harvested by centrifugation at 3000 × *g *for 10 min and the resulting cell pellets treated with Laemmli sample buffer prior to SDS-PAGE on 12% gels. Hemagglutinin yields were estimated by densitometric analysis [26,27] of stained gels following electrophoresis with Quantity One software (Bio-Rad). Spent culture medium was saved for determination of glucose and secreted protein concentration.

### Purification of rHA1

The infected cell pellets were washed once with sodium phosphate buffer (pH 7.4) and then resuspended in lysis buffer (50 mM NaH_2_PO_4_, pH 8.0, 300 mM NaCl, 1% Igepal CA-630) and sonicated in ice for 30 seconds. Soluble and insoluble fractions were separated by centrifugation for 15 min at 12,000 rpm, the insoluble pellet resuspended in Laemmli sample buffer and the proteins resolved by SDS-PAGE. The band corresponding to the 35 kDa rHA1 protein was excised and the protein purified by electroelution (Model 422 Electro-Eluter, Bio-Rad). Purified rHA1 was quantified using a Bradford protein assay kit (Bio-Rad) prior to immunization of guinea pigs.

### Immunoblotting

Estimation of molecular weight and binding properties of hemagglutinin were determined by immunoblotting with minor modifications of the method described by Calandrella et al [28]. First, proteins in each sample were separated by 12% SDS-PAGE and followed by trans-blotting onto nitrocellulose membrane (0.45 μm, Bio-Rad Laboratories, Singapore). The blotting was performed in transfer buffer (25 mM Tris and 192 mM glycine in 20% methanol) at 100 V for 60 min. After blotting, the membrane was blocked by incubation in 3 % blocking solution (3 % non-fat dry milk, 0.1 % Tween 20, 150 mM NaCl, 20 mM Tris) for 1 h at room temperature. The blocked membrane was incubated with rabbit H5N1 antiserum solution (1:1000 v/v) for 1 h at room temperature and washed in TBST (20 mM Tris-HCl, 150 mM NaCl, 0.05% Tween 20). Subsequently, the membrane was incubated with goat anti-rabbit IgG alkaline phosphatase conjugate (1:1000 v/v, Sigma) for 1 h at room temperature. After three washes with TBST, a substrate solution containing 20 ml of 0.1 M Tris-HCl, pH 7.4, 20 mg DAB, 10 μl of H_2_O_2 _was added. After developing the band, the membrane was washed with water to remove the substrate solution.

### Determination of cell density, protein and glucose concentration in the culture medium

Cell density was determined by counting under a stereo light microscope (Olympus, Japan) using a Neubaeur haemocytometer (Weber, England). Triplicate counts were carried out in each experiment. Dead cells were distinguished by Trypan Blue (Sigma-Aldrich, MO) uptake [29].

Secreted protein concentration in the fermentation medium was determined according to the method as described by Bradford [30] using bovine serum albumin (BSA) as the reference standard. Glucose concentration was measured using Dygert method [31].

### Immunolabelling of Sf9 cells and recombinant baculovirus infected Sf9 cells

Normal Sf9 cells and infected Sf9 cells were collected from the suspension culture and allowed to attach in the 4 chamber slides at room temperature (RT) for 2 h. The cells were then washed twice with PBS (pH 7.4) and fixed with 99 % absolute ethanol at RT for 30 min. Before immunostaining, cells were rinsed twice with PBS (pH 7.4). A mouse monoclonal antibody specific to the HA1 protein of H5 was produced (monoclonal antibody 4C12, our unpublished data) and used to identify HA1 protein in the cells. After incubation with monoclonal antibody 4C12, cells were washed several times in PBS (pH 7.4) and visualized with the fluorescence isothiocynate-conjugated anti-mouse antibody (DAKO, Denmark). After labelling, cells were washed twice with PBS and the nuclei were stained with DAPI (4,6-diamidino-2-phenylindole). Cells were washed twice with PBS. Finally, cells were embedded with 2–4 drops of immu-mount (Shandon, USA) and cover slips were left for overnight at RT before examination with a laser scanning fluorescence microscope (Carl Zeiss Laser Scanning Microscope, Axiovert 200 M, LSM510META, Germany) with excitation and emission settings appropriate for the dyes used.

### Immunization of guinea pigs

Guinea pigs were supplied from The Centre for Animal Resources at the National University of Singapore. Animals were approximately 4 months of age with an average body weight of approximately 500 g. Two group of guinea pigs were immunized with partially purified insoluble rHA1 and purified denature rHA1 protein. Each group of six guinea pigs was injected intramuscularly with 50 μg of insoluble rHA1 protein homogenized in Freund's complete adjuvant and 50 μg of purified denature rHA1 protein in Freund's complete adjuvant respectively. After two-weeks they received another dose of 50 μg antigen in Freund's complete adjuvant. Serum samples were taken two weeks after the last injection. Sera were tested for neutralizing activity against H5N1 virus by using a microneutralization assay [31] and hemagglutinin inhibition test (HI) [32].

## Authors' contributions

NN carried out the experiments and drafted the manuscript, HQ quantified the baculovirus, SD and YL determined the haemaggination inhibition and virus neutralization titer of the guinea pigs serums. DQ carried out the monoclonal antibodies production. IM assisted with confocal imaging. BJF proofed and assembled the manuscript, LS proofed the manuscript, and JK prepared the recombinant baculovirus, rabbit anti-H5N1 serum and guinea pig anti-rHA1 serum, and proofed the manuscript. All authors read and approved the final manuscript.

## Figures and Tables

**Table 1:** Antibody response of guinea pigs to recombinant hemagglutinin protein rHA1from influenza virus H5N1.

Protein	HI titer	Neutralization titer
rHA1 (insoluble)	≥ 1:160	≥ 1:160
Denatured rHA1	< 1:20	< 1:10
Control	< 1:10	< 1:10
